# Uncertainty and bias in Liggio et al. (2019) on CO_2_ emissions from oil sands operations

**DOI:** 10.1038/s41467-023-40818-5

**Published:** 2023-09-06

**Authors:** Long Fu, Allan H. Legge

**Affiliations:** 1 Resource Stewardship Division, Alberta Environment and Protected Areas, Edmonton, AB Canada; 2 Biosphere Solutions, Calgary, AB Canada

**Keywords:** Climate sciences, Environmental sciences, Energy and society, Atmospheric chemistry

**arising from** J. Liggio et al. *Nature Communications* 10.1038/s41467-019-09714-9 (2019)

In the April 2019 issue, Liggio et al. published an analysis that utilized CO_2_ emission estimates obtained from aircraft flight studies carried out in the Athabasca Oil Sands area in northeastern Alberta in 2013^1^. The authors reported significant differences between CO_2_ emission estimates from their aircraft flight studies compared to the industry-reported emission estimates with the aircraft flight studies implying a significantly higher level of CO_2_ emissions in the region of 17 megatonnes (MT) per year. We suggest that these apparent discrepancies can be explained by the uncertainty and bias associated with the methods and procedures used in the ref. ^1^ analysis. It is essential that these discrepancies be verified as there are potentially significant financial implications from these emission discrepencies—$1.105 billion per year at a rate of $65/tonne.

## Discrepencies and comparisons of CO_2_ emissions from different methods

We acknowledge that Liggio et al. have made a correction to Figure 3 on November 29, 2022 regarding the omission of the correction factor to account for the molecular weight difference between CO_2_ and SO_2_. The revised Figure 3 shows that the flight result is 62% higher than the CO_2_ emission estimates based on measured SO_2_ emissions for Syncrude Mildred Lake (SML). The revised SML upgrader CO_2_ emissions via the SO_2_ approach (9.8 ± 0.9 MT/y) is more comparible to the industry reported emissions (8.2 ± 0.8 MT/y) as opposed to the flight result (15.9 ± 2.1 MT/y).

Another discrepancy is found for total annual Suncor (SUN) flight CO_2_ emission estimates in Figures 2(b) and 3(d). Based on the author provided information (Figure 2 and Figure 3 tabs in the data file “41467_2019_9714_MOESM3_ESM”), the total flight annual CO_2_ emission estimate of 10.6 MT/y for SUN, the sum of orange and gray bars in Figure 3(d), is 18% (1.6 MT/y) higher than the total flight annual emission estimate of 9.0 MT/y presented in Figure 2(b) of ref. ^1^. This indicates a 50% discrepency for the ground-based emission estimates between two different upscaling approaches—mined ore production and NO_x_/SCO (Synthetic Crude Oil) (see more detailed discussion in Supplementary Information).

We note that there is a typographical error in Fig. S4b miss labeling NO_x_ concentration in ppb as ppm, and should be corrected.

## Monthly CO_2_ emission up-scaling using poor NO_x_ CEMS (Continuous Emissions Monitoring System) data without appropriate validation

The justification for the use of NO_x_ CEMS measurements as a surrogate for production in monthly up-scaling is not supported by industry-provided NO_x_ emission inventory. Equation (5) in ref. ^1^ illustrates how NO_x_ CEMS data was used as a surrogate for SCO production in monthly up-scaling, as opposed to SO_2_ used in ref. ^2^. The monthly up-scaled CO_2_ emisisons were further up-scaled to annual emissions based on SCO production. However, according to Syncrude’s 2013 Air Emissions Summary Annual Report, most of the NO_x_ emissions from the stacks were calculated based on either manual stack surveys or fuel emission factors^3^. Only two Feed Heaters have NO_x_ CEMS measurements among the 22 stationary NO_x_ emission sources at the SML site. The total NO_x_ emissions from the two feed heaters was 461 T/y, which is only 2.2% out of 21,020 T/y total SML NO_x_ emissions. The 461 T/y is equivalent to 52.6 kg/h for an annual hourly average NO_x_ emission rate, which is significantly lower than the 90.4 kg/h and 102.2 kg/h monthly averages reported in Liggio et al. for August and September 2013. Further, four out of five NO_x_ CEMS rates during the SML flights in August 2013 were in the range of 63 to 65 kg/h, far below the monthly average of 90.4 kg/h. Given the data gaps and skewed data distribution, it is inappropriate to use NO_x_ CEMS data as the surrogate for monthly up-scaling.

When using a monthly up-scaling factor of 1 for SML flights (removing NO_x_ CEMS data from up-scaling) as a comparison, annual up-scaling using emission intensity and SCO production will result in an annual CO_2_ emission of 17.0 ± 2 MT as oppose to 24.5 ± 3 MT reported in ref. ^1^. Therefore, using NO_x_ CEMS data for monthly up-scaling results in a 44% higher estimate for SML, which is higher than the 30% overall uncertainty claimed by ref. ^1^.

## The challenge of accounting background CO_2_ and storage-and-release (S-R) effects

Background CO_2_ was determined using upwind measurements in ref. ^1^ for box flights and screen flights where available. Constant extrapolation was used for concentrations below the lowest flight altitude. This approach, however, is not robust enough for screen flights when there are major upwind emission sources. Screen flight F6-SUN has an upwind screen flight F6-SML with an upgrader (elevated plumes) emission of 1.02 × 10^6^ kg/h and ground emission of 5.45 × 10^5^ kg/h. Flight F6-SUN, the downwind screen, only captured the top portion of the elevated plume from SML (see Supplementary Information for more details). The ground level emissions (5.45 × 10^5^ kg/h) and the bottom portion of the elevated plumes (ca. 5.1 × 10^5^ kg/h), about 2/3 of emissions from SML, are below the lowest flight altitude. Figure S(b) F6-SUN in ref. ^1^ shows the mixed plumes from SUN and the top portion of the elevated plume from the upwind SML site. It is not clear as to how the upwind background CO_2_ from SML was accounted for, and how the emission estimate of 1.34 × 10^6^ from SUN was derived [i.e., CO_2_ (downwind) – CO_2_ (upwind)].

A recent study by ref. ^4^ evaluated the impact of storage-and-release on the aircraft-based mass-balance method using model simulated SO_2_ concentration with a known emission rate from the Oil Sands facilities^4^. The TERRA (Top-down Emissions Rate Retrieval Algorithm) SO_2_ emission estimates for the flight on August 28, 2013 around SUN [F14 in ref. ^1^] were rejected by ref. ^4^ due to the impact of elevated emissions from SML and the change in atmospheric stability. The resulting net SO_2_ storage contribution was 156%. This result aligns with the CO_2_ information shown in Fig. S1b F14-SUN. There were much higher CO_2_ concentrations at the upwind walls (north and west) compared to the concentrations at the downwind wall (east). According to ref. ^4^, the average wind direction for F14 was from WNW (297^o^) with a wind speed of 3.2 m/s. With such a significant potential influence from the upwind sources, it is difficult to explain the TERRA CO_2_ emission estimate of 1.37 × 10^6^ kg/h for Flight 14 [i.e., CO_2_ (downwind) – CO_2_ (upwind)], while comparing to the TERRA results for other SUN box flights that have much smaller storage-and-release impacts (Flights 10 and 15). The TERRA results discussed above for F6-SUN demonstrate a missed upwind contribution of 68% based on the CO_2_ data. Both F6-SUN and F14 should be removed based on the rationale provided in ref. ^4^.

Since we did not have access to the TERRA code, despite our request, the TERRA results for Flights 14 and 6–SUN may be the result of extreme cases of high CO_2_ background influences and/or unfavorable meteorological conditions. Some other flights in ref. ^1^ also have low wind speed like Flight 14, especially at low altitudes. It would be benefical to compare the TERRA results at the upwind and downwind walls in ref. ^4^ to the TERRA results at the same waals in ref. ^1^. The release of all individual TERRA results at upwind and downwind walls of the flights and associated meteorological conditions would provide the necessary insight to understand the uncertainties and potential biases identified above.

## Summary

The following is a summary of the key takeaway messages:

1. It is essential that emission estimates and the underlying scientific basis of these estimates be consistently validated in real-world settings along with real-world measurements. The fiscal implications of uncertainties and biases in these estimates must not be ignored, as the consequences can be significant.
2. The rationale for choosing a NO_x_-based monthly up-scaling over an SO_2_-based daily up-scaling is not fully supported by the available evidence. The incomplete data and skewed statistical distribution of CEMS NO_x_ data used in the monthly up-scaling created a significant bias for the SML emission estimates (44%).
3. It is critical to select suitable flights and follow consistent scientific methods. The impacts of storage-and-release on CO_2_ emission estimates due to inconsistent emission rates, upwind emission sources and changes in atomospheric stability are significant, and introduce uncertainties higher than claimed by ref. ^1^.
4. Previous studies show that ground-based emission estimates can be significantly impacted as a result of highly variable vertical wind speed profiles and nearby upwind sources^5^. Also, due to the lack of suitable surrogates for validating and up-scaling ground-based emissions, it is difficult to detect and determine the uncertainties mentioned above. The high level of uncertainty must be addressed when this portion of the TERRA model results are used.
5. To improve the quality of emission estimates, reduce the uncertainty of emission estimates and more acturately reflect real-world emissions, ground-based remote sensing or autonomous measurement platforms should be used in conjunction with aircraft flight measurements.

### Supplementary information

Supplementary Information

## Data Availability

The data analyzed here were originally published in ref. ^1^. The industry reported GHG and NO_x_ emissions are provided in the Supplementary Information.

## Code availability

No custom code or mathematical algorithm was used.
