# Supplementary material for: Uncertainty and bias in Liggio et al. (2019) on CO2 emissions from oil sands operations
Source: Nat Commun. 2023 Sep 6;14:5406. doi: 10.1038/s41467-023-40818-5 (PMC10482826; doi:10.1038/s41467-023-40818-5)
Supplement: Supplementary file 1 — Supplementary Information [file 41467_2023_40818_MOESM1_ESM.pdf]

Supplementary Information

Uncertainty and bias in Liggio et al. (2019) on CO_2 emissions from Oil Sands operations

Long Fu et al.

Classification: Protected A

Acronyms:

TERRA - Top–down Emissions Rate Retrieval Algorithm

SML - Syncrude Mildred Lake

AU - Aurora

SUN – Suncor

GHG - greenhouse gas

CEMS – Continuous Emissions Monitoring System

SCO – Synthetic Crude Oil

Generally accepted definitions on the terminology used:

Scientific uncertainty generally means that there is a range of possible values within which the true value of the measurement lies. Further research on a topic or theory may reduce the level of uncertainty or the range of possible values.

Bias means the systematic or persistent distortion of a measurement process which causes error in a direction. Bias is determined by estimating deviation from the true value as a percentage of the true value.

Generally with source measurements, the true value of a parameter, is rarely known. Instead, environmental agencies commonly use the term "accepted" true value. For example, the US EPA considers that the "true" value is assumed to be that value determined by the US EPA Reference Method.

The discrepancy between Figure 2 and Figure 3 for SUN total annual CO_2 emissions in Liggio et al. (2019)[1]

Supplementary Table 1 below provides supporting information for Figure 3d. in Liggio et al. (2019).

Supplementary Table 1. Upgrading and Ground Based CO_2 emissions from SML and SUN

**Figure 3d:**						
	**Emissions (MT)**					
	**Upgrading CO_2 (Via SO_2 approach)**		**Upgrading CO_2 (Via TERRA approach)**		**Ground Based CO_2 (Via TERRA approach)**	
**SML**	14.2	+/- 1.3	15.9	+/- 2.1	8.1	+/-1.0
**SUN**	6.4	+/- 0.4	5.8	+/- 0.9	4.8	+/-1.0

Supplementary Table 2 below provides emission information extracted from data file “41467_2019_9714_MOESM3_ESM” and the calculated Elevated/Ground emission ratios.

Supplementary Table 2. CO_2 emissions associated with elevated and ground plumes and their ratios for SUN Flights 10 and 15.

Flight	Elevated Plum Only (kg/hr)	Ground Sources Only (kg/hr)	E/G Ratio
10-box	$6.65X10^5$	$4.46X10^5$	1.49
15-box	$7.16X10^5$	$3.32X10^5$	2.16
Average	$6.91X10^5$	$3.89\ X10^5$	1.83

Based on the author-provided data in Supplementary Table 1 above, the total CO_2 emission for SUN in Figure 3d is 10.6 MT (5.8+4.8), which is 18% (1.6 MT) higher than the TERRA derived total annual emission of 9.0 MT shown in Figure 2b in Liggio et al. (2019)[1].

The same hourly TERRA results from SUN flights were used in different up-scaling approaches (Figure 2b – CEMS NO_x data and SCO production, Figure 3d – mined ore) to generate annual CO_2 emission estimates. When a consistent up-scaling approach is used, the resulting annual upgrader vs. ground-based emission ratio should be close to the original hourly emission ratio of 1.83. This ratio would lead to an annual ground-based CO_2 emission of 3.2 MT out of a total of 9.0 MT in Figure 2b. This shows a 50% discrepancy between ground-based CO_2 emissions using different up-scaling approaches as mentioned above

<u>The industry reported CO_2 emissions associated with elevated and ground-based plumes</u>

The following table has SML-AU (Syncrude Mildred Lake – Aurora), and SUN (Suncor) reported GHG in CO_2 equivalent and CO_2 emissions in 2013 (MT/y)

Supplementary Table 3. GHG emissions reported by SML-AU and SUN for 2013 in CO_2 equivalent, MT/y

	SML-AU	SUN
Total reported GHG	12.38	8.41
Total reported CO_2	10.81	7.97
GHG emission from Stack	8.04	6.39
GHG emission from Flaring	0.02	0.20
GHG emission from venting	0.20	0.003
GHG fugitive emission	1.72	0.62
GHG emission from industrial process	1.42	0.55
GHG on-site emission	0.96	0.65
Total CH_4 emission	1.40	0.40
Total N_2O emission	0.16	0.04
CO_2 emission associated with the elevated plume*	8.17	6.58

CO_2 emission associated with the ground-based* plume	2.65	1.39

The above data was extracted from the Environment and Climate Change Canada report:

https://www.canada.ca/en/environment-climate-change/services/climate-change/greenhouse-gas-emissions/facility-reporting/data.html

* The CO_2 emissions associated with the elevated plume is the sum of the stack and flaring emissions plus 50% of venting emission. The CO_2 emissions associated with ground-based plume is the sum of the rest of the GHG emissions less the CO_2 equivalent of CH_4 and N_2O emissions.

Supplementary Table 4. 2013 Syncrude Mildred Lake NO_x Emissions Inventory

Source	ID Number	NO_x Emissions (T/y) as NO_2)	Calculation Method
Main Stack	8F-4	6745	Manual Stack Survey
FGD Stack	26-1F-1	1226	Manual Stack Survey
Utilities Gas Turbines and OTSGs	31GTG-201/202<br>31F-101/102	973	Manual Stack Survey
Bitumen Stacks	21F-7/8/9/10/50/51/52/53	62	Fuel emission factor
Extraction Boilers	31F-46/47	128	
Bitumen Column Feed Heater Stacks	7-1/2F-1A/B	555	Fuel emission factor
Fractionator Feed Heater Stack	7-3F-1	41	NOx CEMS
Steam Superheater Stacks	8-1/2F-6A/B	71	Fuel emission factor
8-3 Steam Superheater Stacks	8-3F-2A/2B	16	Manual Stack Surveys
Reformer Furnace Stacks	9-1/2/3F-1	1243	Fuel emission factor

Plant 9 PSA Tail Gas	9-3F-1	172	Fuel emission factor
Hydrogen Reformer Feed Heater Stack	9-4F-1	420	NOx CEMS
Plant 15 Hydrogen Heater Stacks<br>Plant 15 Reboiler Stacks	15-1/2F-1<br>15-1/2F2	81<br>54	Fuel emission factor
Plant 18-1 Stacks	18- 1F-1/2	46	Fuel emission factor,<br>Manual Stack Survey
Plant 18-2 Stacks	18-2F-1/2	27	Manual Stack Survey
Plant 22 Stacks	22-1F-1	29	Fuel emission factor
Plant 37-1 Stacks	37-1F-1/2	33	Manual Stack Survey
Diluent Reboiler Stack	14F-1	26	Fuel emission factor
Sulfreen Regeneration Furnace Stack	12-0F-101	8	Fuel emission factor
Flared Gas	19F36/37/38	177	Fuel emission factor
Mine Diesel Equipment		7233	Fuel emission factor
LPNG System Furnace Pilots		327	Fuel emission factor
Mine Service Building Building Heating		33	Fuel emission factor
Other*		1284	Fuel emission factor
Gasoline and Propane		8	Fuel emission factor

Biomass Combustion		0	Fuel emission factor
**Total NOx (T/y) as NO2**		**21,020**	
**Total NOx (T/d) as NO2**		**57.43**	

* Emissions assigned to the discrepancy between total fuel in (natural gas, refinery fuel gas, purge gas) and gauge fuel consumed.
The above information was extracted from the Syncrude Air Emissions Summary 2013 Annual Report.

Supplementary Table 5 2013 Syncrude Mildred Lake NO_x emission from Feed Heater stacks, and SO_2 emission from Flue-gas desulphurization (FGD) Operation for August, September and annual emission everages.

Month	SO_2 (% Recovery)	SO_2 Emission (T/d)	NO_x Emission (kg/hr)
August	93.81[a]	11.74[a]	90.4[b]
September	93.71[a]	10.87[a]	102.2[b]
Annual Average	92.25[a]	13.63[a]	52.2[a]

The above information was extracted from (a) the Syncrude Air Emissions Summary 2013 Annual Report and (b) data file "41467_2019_9714_MOESM3_ESM") provided by Liggio et al. (2019)[1].

<u>The Impact of Significant Upwind Emission Sources for F6-SUN</u>

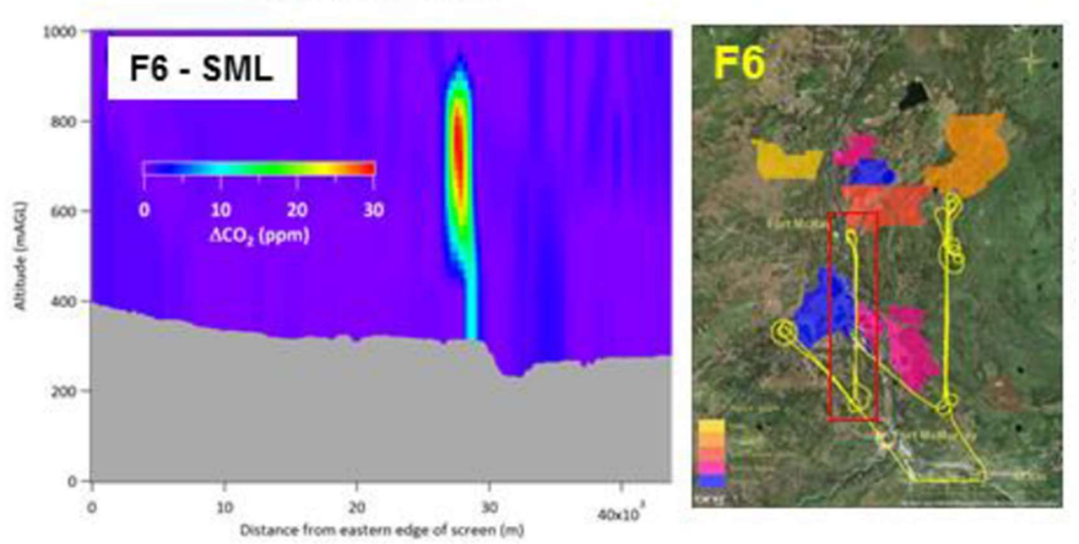

Supplementary Figure 1. Image illustrating the upwind screen Flight 6 for SML in Liggio et al. (2019). Map data: Bing Maps, 2015[1].

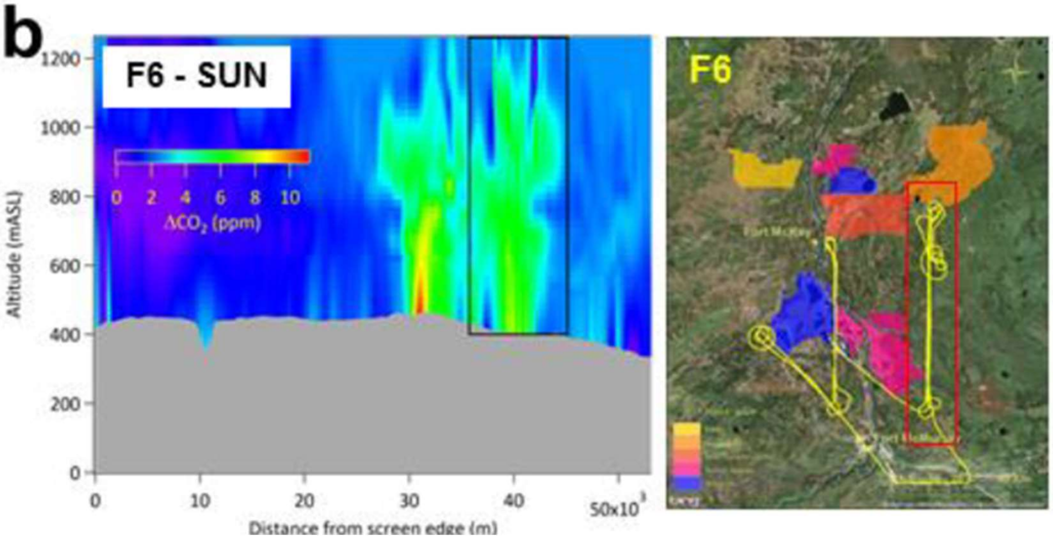

Supplementary Figure 2. Image illustrating the downwind screen Flight 6 for SUN in Liggio et al. (2019). Map data: Bing Maps, 2015[1].

The upwind F6-SML screen flight captured both elevated and ground-based plumes, with a vertical dimension of about 400 meters and the highest ΔCO_2 level of 30 ppm for the elevated plume. The downwind F6-SUN screen only captured the top portion of the elevated plume from SML. The vertical dimension of the top portion of the elevated plum is about 800 meters and the highest ΔCO_2 level is about 10 ppm. There is no specific description if the upwind screen information from F6-SML was used in calculating the F6-SUN emissions. The upwind emissions from SML would have significant impacts on extrapolation and total CO_2 emission estimates. This is because the SML plumes and SUN plumes are mixed with similar ΔCO_2 levels in the downwind screen.

Assuming 50% of the elevated plume from SML is below the lowest flight altitude in the F6-SUN screen, the estimated net uncounted contribution from SML would be about 68%, which is significantly higher than the 30% overall uncertainty claimed by Liggio et al. (2019)[1].

<u>The CO_2 equivalent impact of Storage-and-Release on Emission Estimate for Flight 14</u>

Fathi et al. (2021)[2] determined a 156% storage impact for F14 using the simulated ambient SO_2 data and attributed this impact to the upwind emissions from SML. Given the average $\Delta CO_2/\Delta SO_2$ ratios of 225 and 466 for SML and SUN, respectively, the CO_2 equivalent net storage contribution is about 75% [(225X1.56)/466]. It is recognized that $\Delta CO_2/\Delta SO_2$ ratios from SML and SUN for August 28, 2013 would have been better suited for this calculation, if available.

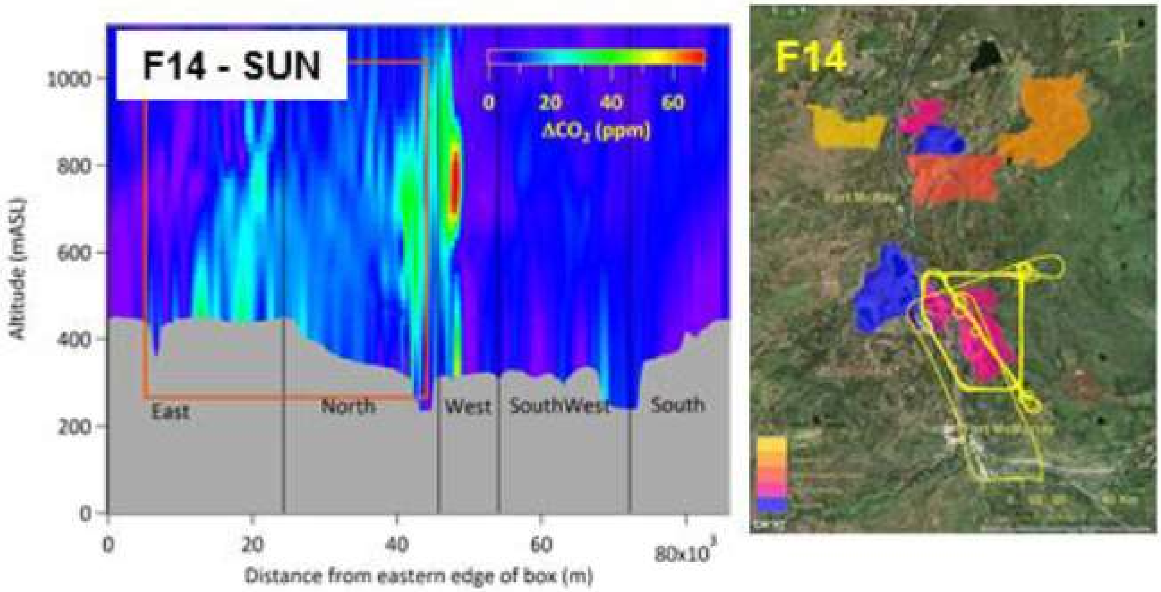

Supplementary Figure 3. Image illustrating the box Flight 14 walls for SUN in Liggio et al. (2019). Map data: Bing Maps, 2015[1].

For Flight 14, the average wind direction was from WNW (297°) with a wind speed of 3.2 m/s according to Fathi et al. (2021)[2]. The Supplementary Figure 3 for F14 – SUN above shows a much higher CO_2 concentration at the upwind walls (North and West, 40 – 60 ppm) compared to the concentration at the downwind wall (East, 20 – 40 ppm).

Data uncertainties

Uncertainties associated with the data from Liggio et al. (2019)[1] were provided by the authors in their Supplementary Information.

An uncertainty of 10% is assumed for industry reported emission inventory data. The same uncertainty is used for the recalculated emissions using industry reported emissions data.

Aligned with the approach used in the Liggio et al. (2019)[1], a 10% uncertainty is used for the recalculated emission estimates under various up-scaling scenarios.

Supplementary References:

1. Liggio, J. et al. Measured Canadian oil sands CO_2 emissions are higher than estimates made using internationally recommended methods. Nat. Commun. 10, 1863 (2019), https://doi.org/10.1038/s41467-019-09714-9.

2. Fathi, S. et al. Evaluating the impact of storage-and-release on aircraft-based mass-balance methodology using a regional air-quality model. Atmos. Meas. Tech. 8, 3745-3765 (2021), https://doi.org/10.5194/acp-21-15461-2021.

Classification: Protected A
